# Investigation of viscerotropism-associated genes in *Leishmania tropica* strains causing visceral leishmaniasis

**DOI:** 10.3389/fmicb.2026.1831849

**Published:** 2026-05-11

**Authors:** Ahmet Ozbilgin, Aslı Tetik Vardarlı, Ibrahim Cavus, Melike Dinc, Varol Tunali, Merve Beyaz, Sukran Kose, Talat Yalcin, Cumhur Gündüz

**Affiliations:** 1 Department of Parasitology, Faculty of Medicine, Celal Bayar University, Manisa, Türkiye; 2 Department of Medical Biology, Faculty of Medicine, Ege University, Izmir, Türkiye; 3 Department of Chemistry, Izmir Institute of Technology, Izmir, Türkiye; 4 Department of Emergency Medicine, Eşrefpaşa Municipality Hospital, Izmir, Türkiye; 5 Department of Infectious Diseases and Clinical Microbiology, Faculty of Medicine, Dokuz Eylul University, Izmir, Türkiye

**Keywords:** cutaneous leishmaniasis, *Leishmania tropica*, NGS, virulence, visceral leishmaniasis

## Abstract

*Leishmania infantum* is the predominant etiological agent of visceral leishmaniasis (VL), whereas *Leishmania tropica* is classically associated with cutaneous leishmaniasis (CL). However, sporadic reports from Turkiye and other endemic settings indicate that *L. tropica* may also cause VL, suggesting that specific parasite determinants enable adaptation to visceral tissues. The molecular basis of this phenotypic shift remains insufficiently understood. This study aimed to define genetic factors associated with visceralization in *L. tropica* by integrating clinical isolates from patients with VL and CL and evaluating candidate genes involved in oxidative stress defense, mitochondrial function, proteolysis, and metabolic adaptation. Fourteen patients diagnosed between 2012 and 2022 were included, comprising seven VL and seven CL cases. Parasites isolated from clinical specimens were cultured and genotyped as *L. tropica* using real-time quantitative PCR targeting the internal transcribed spacer 1 region. Gene-expression profiling by qRT-PCR focused on Cytochrome C Oxidase subunit IV, Metallo-peptidase (Clan MA(E), Family M32), Oligopeptidase B, Peroxiredoxin 1, Peroxiredoxin 2, Pyruvate kinase, and Succinyl-CoA:3-ketoacid-coenzyme A transferase. Compared with CL isolates and reference strains, VL isolates demonstrated markedly increased mRNA expression of Peroxiredoxin 1 and Peroxiredoxin 2 and Cytochrome C Oxidase subunit IV, with approximately 17-fold and 21-fold elevations, respectively. Additional increases were observed in metallo-peptidase, Oligopeptidase B, and Succinyl-CoA:3-ketoacid-coenzyme A transferase, supporting a broader program of antioxidant, mitochondrial, proteolytic, and metabolic adaptation. Targeted next-generation sequencing identified multiple coding variants in Oligopeptidase B and the M32 metallo-peptidase, suggesting potential contributions to phenotypic divergence between VL and CL isolates. Viscerotropic *L. tropica* exhibits a distinct molecular profile characterized by enhanced antioxidant defense, mitochondrial activity, proteolytic capacity, and metabolic flexibility. These findings identify plausible genetic drivers of visceralization, expand current understanding of *L. tropica* pathogenicity, and support the development of testable diagnostic and therapeutic targets. Collectively, the data challenge the prevailing view of *L. tropica* as an exclusively cutaneous parasite. The results also provide a rational framework for future functional validation studies aimed at clarifying causality, refining biomarkers, and prioritizing parasite specific intervention strategies in endemic settings globally.

## 1 Introduction

There are approximately 30 distinct *Leishmania* species known to infect mammals; 10 species are identified in the Old World, whereas the remaining 20 species are present in the New World. *Leishmania* parasites exhibit a digenetic life cycle comprising two distinct morphological stages: extracellular promastigotes within the invertebrate vector, and intracellular amastigotes residing in the macrophages of vertebrate hosts. Of these, 21 species have been identified as pathogenic to humans (Stephenson, 2007; Alvar et al., 2012; McCall et al., 2013). Leishmaniasis, a disease caused by intracellular parasites, is endemic primarily in tropical and subtropical regions. It is transmitted predominantly by the bite of infected female sand flies belonging to the genera Phlebotomus and Lutzomyia, prevalent across Europe, North Africa, the Middle East, Asia, and parts of Central America. Recognized by the World Health Organization (WHO) as one of the seven most significant neglected tropical diseases, leishmaniasis constitutes a major public health concern due to its high morbidity, diverse clinical presentations, and considerable mortality. Endemic areas include Central and South America, parts of Southeast Mexico, Southern Europe, the Middle East, Africa, and Asia (Arenas et al., 2017). Leishmaniasis disproportionately affects impoverished populations, and its incidence is closely associated with socioeconomic factors such as malnutrition, population displacement, inadequate housing, compromised immunity, and limited healthcare resources. As of 2022, among the 200 countries and territories reporting to WHO, 99 were classified as endemic for leishmaniasis; of these, 71 countries reported both visceral and cutaneous leishmaniasis, nine reported exclusively visceral leishmaniasis (VL), and 19 reported exclusively cutaneous leishmaniasis (CL). Approximately 85% of global VL cases originated from Brazil, Ethiopia, India, Kenya, Somalia, South Sudan, and Sudan, while eight countries—Afghanistan, Algeria, Brazil, among others—accounted for 85% of global CL cases (Clinical Care of Leishmaniasis | Leishmaniasis | CDC, n.d.).

Clinical manifestations of *Leishmania* infections include visceral leishmaniasis, cutaneous leishmaniasis, and mucocutaneous leishmaniasis (MCL), each correlated with distinct *Leishmania* species and characteristic clinical symptoms. VL, primarily caused by *Leishmania donovani* and *Leishmania infantum*, is characterized clinically by prolonged fever, hepatosplenomegaly, significant weight loss, anemia, leukopenia, and thrombocytopenia. CL, most commonly associated with *Leishmania major* and *Leishmania braziliensis*, typically presents with localized nodular lesions that may ulcerate. The severity and progression of CL lesions are heavily influenced by the host’s immune response. MCL, predominantly linked to *L. braziliensis* infection, involves extensive mucosal damage and severe morbidity. Although VL represents the most severe and potentially fatal form, CL remains more prevalent, particularly in endemic regions. Accurate diagnosis is complicated by clinical presentations overlapping with other diseases, underscoring the necessity for precise diagnostic methodologies (Bailey and Lockwood, 2007; Mathers et al., 2007; Pedrosa and Pedrosa, 2017; Scorza et al., 2017; Kumar et al., 2023).

Recent studies have indicated that certain *Leishmania* species previously believed to cause exclusively cutaneous or visceral disease may, in fact, be capable of inducing both clinical forms (Burza et al., 2018; Thakur et al., 2018). Nevertheless, significant evidence demonstrates clear species-level divergence between cutaneous and visceral strains, notably regarding their distinct adaptations for visceral tropism (McCall et al., 2013). These adaptations include tolerance to higher temperatures found within internal organs (Callahan et al., 1996), enhanced resistance to oxidative stress, a crucial aspect of host immune defense (Sarkar et al., 2012) and differential host cell tropism (De Trez et al., 2009). Additionally, parasite, host, and vector factors collectively influence visceralization, with the A2 gene family notably implicated as essential for establishing visceral infection (Charest and Matlashewski, 1994; Zhang and Matlashewski, 1997; Mizbani et al., 2011).

The genus *Leishmania* is characterized by a meiosis-like reproductive mechanism, contributing significantly to intraspecific genetic variant (Inbar et al., 2019). Specifically, *Leishmania tropica* exhibits remarkable genomic plasticity, frequently undergoing genetic exchange events, including sexual reproduction, at rates higher than observed in other *Leishmania* species. Observed intra- and inter-chromosomal rearrangements in *L. tropica* suggest active regulatory processes involving mitotic, meiotic, and parasexual mechanisms (Glans et al., 2021). The extensive genetic diversity observed in *L. tropica* is attributed to its digenetic life cycle, inherent chromosomal instability, frequent hybridization events, and high allelic variability. Such factors collectively enable rapid adaptation and increased pathogenic potential, complicating disease control and therapeutic intervention strategies (Iantorno et al., 2017; Bussotti et al., 2021; Glans et al., 2021). Despite knowledge of tropism toward either cutaneous or visceral involvement, the underlying molecular mechanisms remain incompletely understood (Ait Maatallah et al., 2022). In this study, the following research questions and objectives were defined: What are the potential genetic determinants associated with visceralization in *L. tropica* isolates? Is there a relationship between the expression levels of the identified genes and the clinical course of the disease? What is the impact of genetic variations on parasite pathogenicity? The primary objective of this research is to identify genetic markers specific to visceralization in *L. tropica* strains isolated from immunocompetent hosts, thereby contributing to a deeper understanding of the molecular mechanisms underlying parasite pathogenicity and clinical outcomes.

## 2 Materials and methods

### 2.1 Patients

This study analyzed promastigotes isolated and cultured from 14 individuals with confirmed *Leishmania tropica* infection, including seven patients with visceral leishmaniasis (VL) and seven with cutaneous leishmaniasis (CL), to investigate genetic determinants associated with viscerotropism. Inclusion criteria were defined as follows: VL cases presented with clinical manifestations consistent with visceral disease, including fever, hepatomegaly, splenomegaly, and pancytopenia. CL cases presented with persistent cutaneous lesions of at least 2 months’ duration. Exclusion criteria were applied to ensure clear phenotypic separation between groups: for the VL group, the presence of any cutaneous lesion was an exclusion criterion; for the CL group, systemic manifestations suggestive of visceral involvement (e.g., hepatosplenomegaly, pancytopenia, and fever) constituted exclusion criteria. All participants were diagnosed and treated at the Parasitology Laboratory, Faculty of Medicine, Manisa Celal Bayar University (Türkiye), between 2012 and 2022. Diagnostic confirmation was based on parasitological evidence in addition to clinical presentation. For VL, bone marrow aspirates were examined by light microscopy after Giemsa staining, and the presence of amastigote forms was used as a direct parasitological criterion. In parallel, bone marrow specimens were cultured on Novy–MacNeal–Nicolle (NNN) medium, and the emergence of promastigote forms provided culture confirmation. Serological testing was performed by the indirect fluorescent antibody test (IFAT), and VL cases demonstrated seropositivity (typically ≥1:256). For CL, diagnosis was confirmed by demonstration of amastigotes in Giemsa-stained smears prepared from lesion scrapings and by positive culture from lesion material on NNN medium with promastigote outgrowth. Parasite genotyping was systematically performed to confirm species identification as *L. tropica*. Patients with VL received intravenous liposomal amphotericin B at 3 mg/kg/day on days 1–5, with additional doses on days 14 and 21 (total of seven doses). Clinical improvement—defined as resolution of fever, normalization of liver and spleen size, and recovery of hematological parameters—was observed by the end of the first month of therapy. Abdominal ultrasonography further confirmed regression of hypoechoic splenic nodules. Patients were discharged after full clinical recovery and were followed through routine outpatient visits. Patients with CL were treated with intralesional therapy administered twice weekly. At least eight injections were delivered directly into the lesion(s) and continued until complete blanching of the lesion(s) was achieved (Clinical Care of Leishmaniasis | Leishmaniasis | CDC, n.d.).

### 2.2 Clinical sample collection and parasite culture

Preliminary diagnoses of VL were confirmed by analyzing clinical specimens collected via fine needle aspiration from pelvic bone marrow by authorized physicians. For CL cases, the lesion area and surrounding healthy skin were first cleansed with 70% ethanol, followed by injection of 0.2–0.5 mL sterile saline solution directly into the lesion. Amastigotes were identified microscopically after staining the aspirate samples with Giemsa stain. All clinical samples were cultured initially on nutrient-enriched NNN medium supplemented with cow milk and cow liver extract (EM medium), as previously described (Özbilgin et al., 2016). Promastigote cultures were incubated at 26 °C and monitored daily for 1 month. Subsequently, established promastigote cultures were transferred to RPMI 1640 medium supplemented with 10% fetal calf serum (FCS), 200 U/mL penicillin, and 0.2 mg/mL streptomycin, and expanded in 25 mL flasks (5 mL culture volume) to yield sufficient quantities for genomic and gene expression analyses. Parasite density was adjusted to 1 × 10^8^ cells/mL, washed five times in sterile saline, and utilized in subsequent experiments.

### 2.3 *Leishmania* genotyping

Genotyping of amastigotes obtained from the clinical specimens of patients and promastigotes isolated from NNN medium were performed using real-time PCR assays targeting the ribosomal internal transcribed spacer 1 (ITS1) region, which separates the genes encoding the small subunit (SSU) and 5.8S ribosomal RNAs in *Leishmania* species. DNA isolation from all isolates and clinical specimens was performed using the High Pure PCR Template Preparation Kit (Roche). Primers for amplification were ITS1 Forward (5′-CTGGATCATTTTCCGATG-3′) and ITS1 Reverse (5′-GAAGCCAAGTCATCCATCGC-3′), utilizing the QuantiTect Probe PCR Master Mix. Species differentiation was achieved by melting curve analysis employing specific fluorescent probes: Probe 1 (5′-CCGTTTATACAAAAAATATACGGCGTTTCGGTTT-Fluo-3′) and Probe 2 (5′-LCRed640-GCGGGGTGGGTGCGTGTGTG-Pho-3′). The thermal profile applied to differentiate between *Leishmania* species (*L. tropica, L. infantum, L. donovani*, and *L. major*) consisted of denaturation, amplification, melting curve analysis, and cooling steps. Genotyping of the analyzed strains was performed based on melting curve analysis, using the melting profiles of reference strains as a comparative standard. Reference strains included in genotyping were *L. tropica* (MHOM/AZ/1974/SAF-K27), *L. major* (MHOM/SU/1973/5ASKH), *L. infantum* (MHOM/TN/1980/IPT1), and *L. donovani* (MHOM/IN/1980/DD8) (Toz et al., 2013).

### 2.4 Real-time qRT-PCR analysis

Expression levels of seven previously identified genes associated with viscerotropism (Dinç, 2018)—Peroxidoxin 1 and 2, Oligopeptidase B, Metallo-peptidase (Clan MA(E), Family M32), Cytochrome C Oxidase subunit IV (COX4), Succinyl-CoA:3-ketoacid-coenzyme A transferase, and Pyruvate kinase—were quantified at the mRNA level. Promastigotes were selected for gene expression analyses due to their ease of culture and suitability for standardized laboratory conditions. Although amastigotes represent the pathogenic intracellular stage in human hosts, technical limitations associated with their consistent *in vitro* cultivation necessitated using promastigotes as an experimental model to investigate genes potentially involved in viscerotropism. Promastigotes from primary NNN cultures exhibiting growth within 5–6 days were subcultured into RPMI-1640 medium and utilized at the logarithmic growth phase. Cultured parasites were cryopreserved without undergoing a second passage. Total RNA was extracted from promastigotes (3 × 10^7^ cells) of seven CL, seven VL, and reference *L. tropica* strains (MHOM/AZ/1974/SAF-K27) and *L. infantum* strains (MHOM/TR/2006/CBU20) using TRIzol reagent (Invitrogen) according to the manufacturer’s instructions. Complementary DNA (cDNA) synthesis was conducted using SuperScript II Reverse Transcriptase (Invitrogen), followed by purification with the QIAquick PCR purification kit (Qiagen), following the manufacturer’s guidelines. Real-time quantitative reverse transcriptase PCR (qRT-PCR) assays were performed utilizing QuantiTect SYBR Green PCR Kit (Qiagen) with gene-specific primers (second primer pairs for each gene provided in Supplementary Table S1) on the Rotor-Gene Q instrument (Qiagen). Amplification conditions were as follows: an initial denaturation step at 95 °C for 15 min, followed by 45 cycles consisting of denaturation at 95 °C for 30 s, annealing at 55 °C for 30 s, and extension at 72 °C for 30 s. Gene expression normalization was performed using 18S rRNA as the internal control, and relative fold-changes were calculated using the 2^−ΔΔCt^ method in comparison to reference *L. tropica* gene expression. PCR amplification efficiencies for all target genes and the 18S rRNA reference gene were calculated using standard curves and were found to be comparable, supporting the use of the 2^ΔΔCt^ method for relative quantification. All samples were analyzed in triplicate.

### 2.5 Evaluation of genes by comparing gene sequences

Next,-generation sequencing (NGS) was employed to sequence gene regions hypothesized to be responsible for viscerotropism, using genomic DNA (gDNA) derived from *L. tropica* isolates obtained from seven visceral leishmaniasis and seven cutaneous leishmaniasis patients. NGS provides deep sequencing coverage (≥100 reads per region), facilitating the detection of low-frequency single nucleotide polymorphisms (SNPs) and copy number variations (CNVs). Although the *L. tropica* genome assembly remains incomplete, relevant gene sequences are accessible via publicly available genome databases.

Genomic DNA was extracted from seven VL and seven CL isolates using a commercial genomic DNA extraction kit (Thermo Scientific) according to the manufacturer’s instructions. For sequencing on the Ion Torrent PGM platform, approximately 100 ng of gDNA per isolate was utilized. Targeted gene regions were amplified using specific primers listed in Supplementary Table S1. Following amplification, fragment libraries were prepared using the Ion Xpress Plus Fragment Library Kit, following the manufacturer’s protocol (de Oliveira et al., 2017).

Barcoding of each *Leishmania* isolate library was achieved through ligation of Ion Xpress Barcode adapters to the fragmented DNA, according to the kit guidelines. Library normalization prior to template preparation was performed using the Library Equalizer Kit. Libraries were subsequently amplified using emulsion PCR on Ion Sphere Particles (ISPs) via the Ion PGM Hi-Q OT2 Kit, according to the manufacturer’s recommendations (Rao et al., 2016). Enrichment of templated ISPs was performed with the Ion PGM Enrichment Beads kit. Quality control assessments of enriched ISPs were conducted using the Ion Sphere Quality Control Kit before sequencing. Sequencing was performed on an Ion 316 Chip Kit, enabling ISPs to be sequenced across millions of wells by the Ion PGM sequencer.

Data analysis was performed using the Torrent Suite software, where base sequences were evaluated, and genetic variations between the *L. tropica* strains were identified with the Variant Caller software integrated into the CLC Genomics Workbench platform. The Variant Caller software identifies highly specific genetic variants using a fixed ploidy model (ploidy set to 2, variant probability threshold ≥50%), employing a Bayesian statistical approach combined with maximum likelihood analysis. Identified gene variants were compared against reference sequences from known VL-causing *Leishmania* species to evaluate their biological significance and relevance (Šlapeta et al., 2015). Reads were aligned to target gene regions, and variant analyses were performed to detect mutations.

Additionally, gene expression levels correlating with identified genetic mutations were quantified via quantitative real-time PCR (qRT-PCR) analysis using total RNA extracted from the same *Leishmania* isolates, as described in Section 2.4.

### 2.6 Statistical analysis

Statistical comparisons among the three experimental groups were conducted using one-way analysis of variance (ANOVA) following confirmation of data normality through appropriate tests. All statistical analyses were performed using GraphPad Prism software version 9.5. Differences were considered statistically significant at a *p*-value of < 0.05.

## 3 Results

### 3.1 Leishmaniasis cases

**Figure 1:**
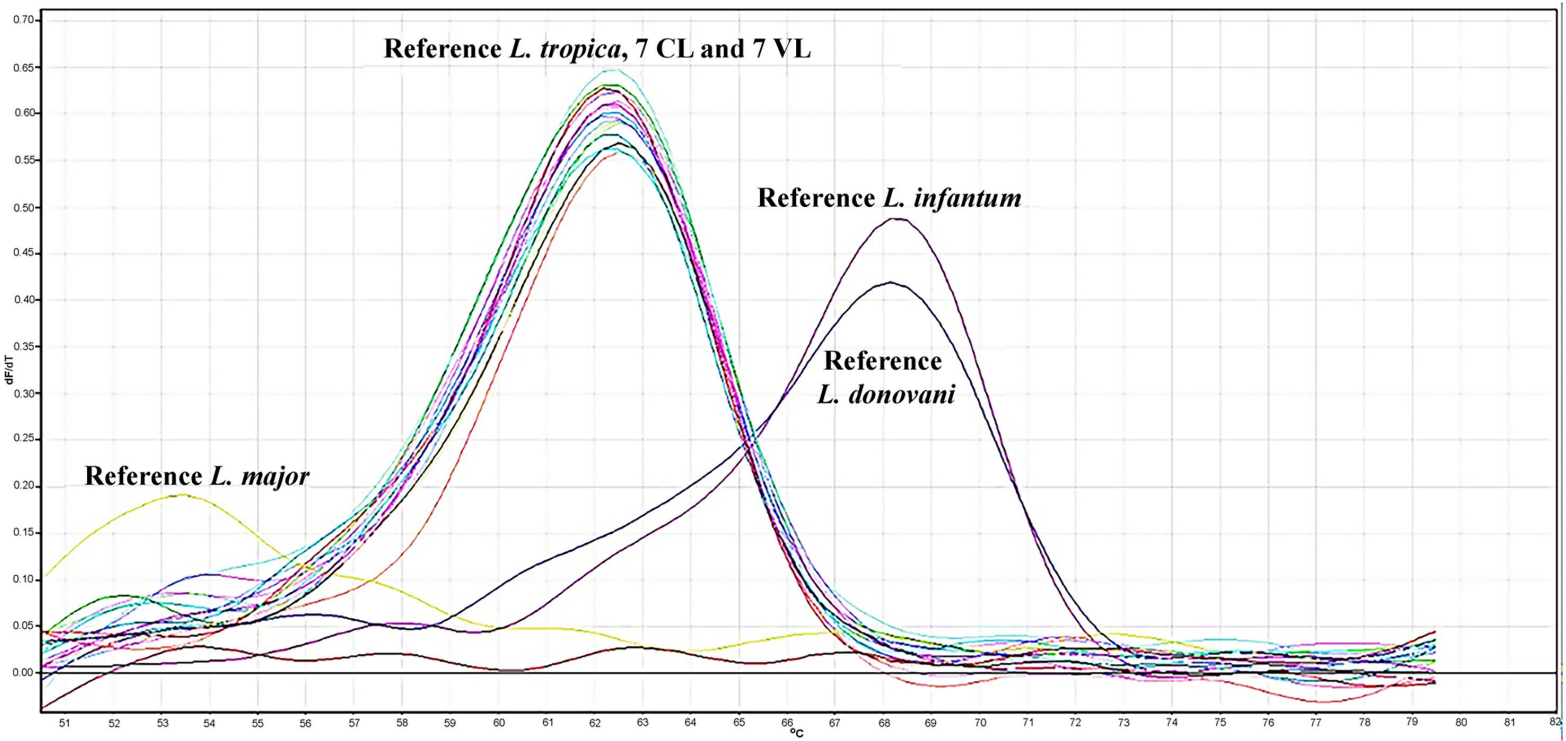
Melting curves from quantitative polymerase chain reaction (qPCR) analyses of clinical specimens and promastigote cultures derived from leishmaniasis patients. Genotyping was performed using reference strains: *Leishmania tropica* (MHOM/AZ/1974/SAF-K27), *Leishmania major* (MHOM/SU/1973/5ASKH), *Leishmania infantum* (MHOM/TN/1980/IPT1), and *Leishmania donovani* (MHOM/IN/1980/DD8).

This study included 14 patients with *Leishmania tropica* infection, comprising seven individuals with visceral leishmaniasis (VL) and seven with cutaneous leishmaniasis (CL), evaluated and treated at the Parasitology Laboratory, Faculty of Medicine, Manisa Celal Bayar University (Türkiye), between 2012 and 2022. Patient-level demographic and geographic information (age, sex, and region), together with the principal clinical presentation and species genotyping results, are summarized in Table 1. Key clinical and laboratory characteristics of the VL cohort are provided in Table 2. Comprehensive clinical assessment indicated that none of the patients in either group had underlying immunological or systemic comorbidities. All CL cases were negative by the rK39 rapid diagnostic (dipstick) test, whereas IFAT results for VL cases were obtained as part of the routine diagnostic work-up in the clinical laboratory (Tables 1, 2). Species identification was confirmed for all 14 isolates by *Leishmania* genotyping using ITS1-targeted quantitative PCR (qPCR) performed on promastigotes derived from the patients’ clinical samples, verifying *L. tropica* in every case (Figure 1; Table 1). All patients achieved complete clinical and parasitological recovery during follow-up, and no evidence of drug resistance was observed. CL patients received intralesional meglumine antimoniate administered every 3 days for a total of eight sessions (continued until complete blanching of lesions). VL patients were treated with intravenous liposomal amphotericin B at 3 mg/kg/day for five consecutive days, followed by additional doses on days 14 and 21 (total of seven doses). In VL cases, clinical improvement—defined by resolution of fever, normalization of spleen and liver size, and recovery of hematological parameters—was documented in all patients, and follow-up ultrasonography confirmed regression of hypoechoic splenic nodules. All patients were discharged after full recovery and were subsequently monitored through routine outpatient visits.

**Table 1:** Clinical histories and *Leishmania* species genotyping results of patients included in the study.

Patient code	Gender	Age	Region	Symptom	Genotype of amastigotes from clinical samples	Genotype of promastigotes grown in culture
V1	M	7	Aegean	Fever, rapid weight loss, hepatosplenomegaly, pancytopenia, nausea, diarrhea	*L. tropica*	*L. tropica*
V2	F	10	Aegean	Weight loss, weakness, fever, anorexia, pancytopenia, nose and tooth bleeding, hepatosplenomegaly, growth retardation	*L. tropica*	*L. tropica*
V3	F	12	Aegean	Swelling in the left upper quadrant, night sweats, anorexia, rapid weight loss (5 kg in the last 1 month), splenomegaly, pancytopenia	*L. tropica*	*L. tropica*
V4	M	20	Aegean	Fever, hepatosplenomegaly, anorexia, spleen infarction, general condition disorder, diarrhea, pancytopenia, nose and gum bleeding, general condition disorder	*L. tropica*	*L. tropica*
V5	M	50	Aegean	Fever, diarrhea, nausea, anorexia, dizziness, weakness, thrombocytopenia, leukopenia, hepatosplenomegaly	*L. tropica*	*L. tropica*
V6	M	53	Aegean	Fever, anorexia, pancytopenia, hepatosplenomegaly, weight loss, malaise, diarrhea	*L. tropica*	*L. tropica*
V7	M	55	Mediterranean	Fever, weight loss, pancytopenia, hepatosplenomegaly, anorexia, nausea	*L. tropica*	*L. tropica*
C1	M	10	Aegean	Nodular, dry-type lesion on the right cheek for 3 months	*L. tropica*	*L. tropica*
C2	M	11	Aegean	A dry-type lesion, characterized by erythematous plaques with prominent vascularity, on the skin of the right zygomatic region of the face for 24 months	*L. tropica*	*L. tropica*
C3	F	17	Aegean	A dry-type cutaneous lesion on the right foot for 3 months	*L. tropica*	*L. tropica*
C4	F	18	Aegean	A dry-type lesion on the right side of the nose for 7 months	*L. tropica*	*L. tropica*
C5	M	25	Aegean	Dry type lesion on the right cheek for 8 months	*L. tropica*	*L. tropica*
C6	F	35	Aegean	A dry-type lesion on the left cheek, located inferior to the eye for 6 months	*L. tropica*	*L. tropica*
C7	F	47	Aegean	A dry-type, pruritic lesion on the tip of the nose for 12 months	*L. tropica*	*L. tropica*

**Table 2:** Clinical and laboratory characteristics of visceral leishmaniasis patients.

Clinical feature	V1	V2	V3	V4	V5	V6	V7
Fever	+	+	+	+	+	+	+
Weight loss	+	+	+	−	+	+	−
Fatigue	+	+	−	+	+	+	+
GIS symptoms	−	+	+	+	−	+	+
Epistaxis/gingival bleeding	+	−	−	−	−	−	+
Splenomegaly	+	+	+	+	+	+	+
Hepatomegaly	+	+	+	+	−	+	+
Pancytopenia	+	+	+	−	+	+	+
Leukopenia	−	−	−	+	−	−	−
Thrombocytopenia	−	−	−	+	−	−	−
Microscopy	Positive	Negative	Positive	Positive	Positive	Positive	Positive
Modified NNN	Positive	Positive	Positive	Positive	Positive	Positive	Positive
Seropositivity (IFAT)	1/512	1/512	1/512	1/1024	1/512	1/1024	1/1024
qPCR (Bone marrow)	Positive	Positive	Positive	Positive	Positive	Positive	Positive

### 3.2 mRNA expression alterations of genes associated with viscerotropism

**Figure 2:**
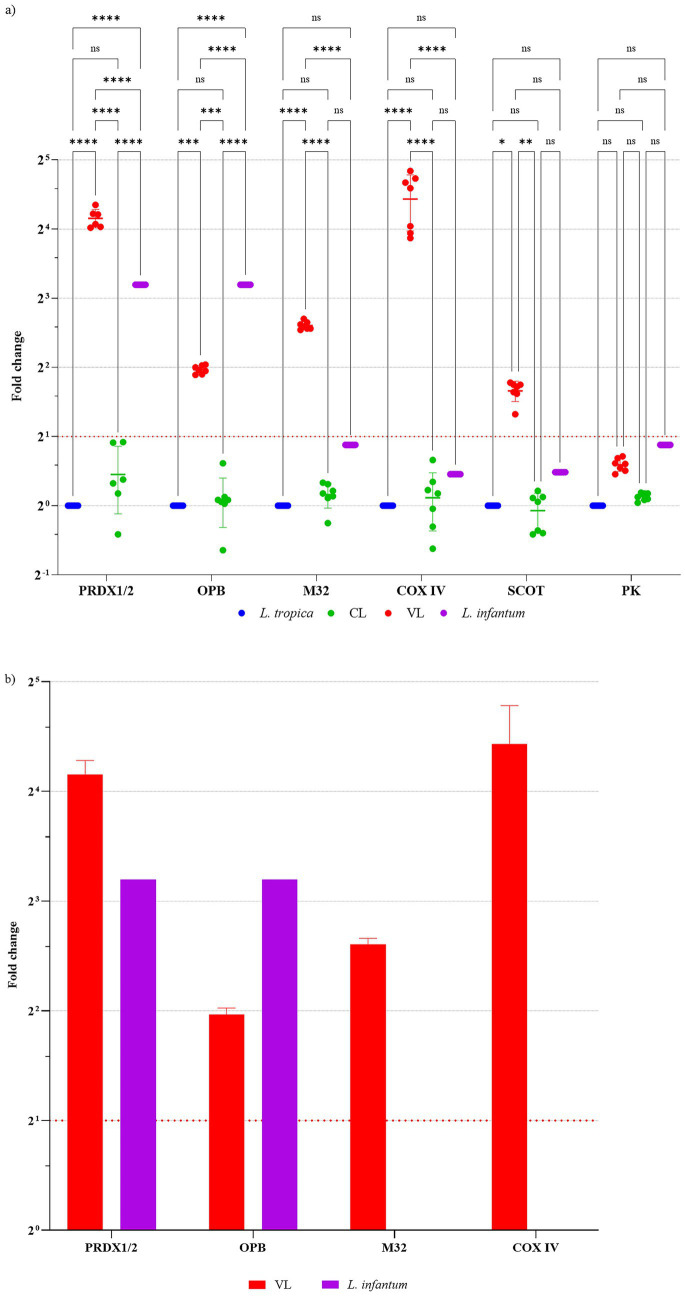
Changes in mRNA expression of genes associated with viscerotropism. **(a)** Relative expression values (2^−ΔΔCt^) are shown as scatter plots for each gene, with each point representing an individual isolate. Group mean and standard deviation (SD) are indicated. **(b)** Bar plots (mean ± SD) are provided only for genes showing statistically significant differences. Because the reference *L. tropica* strain serves as the normalization baseline in the 2^−ΔΔCt^ calculation (set to 1.0), it is not displayed as a bar and is not included in statistical comparisons. Comparison groups include cutaneous leishmaniasis (CL) isolates (*n* = 7), visceral leishmaniasis (VL) isolates (*n* = 7), and the reference *L. infantum* strain. Expression levels were normalized to 18S rRNA and calculated relative to reference *L. tropica* (MHOM/AZ/1974/SAF-K27) = 1.0. Peroxiredoxin-1 and Peroxiredoxin-2 are reported jointly (PRDX1/2) due to high sequence homology. Group differences for each gene were tested using one-way ANOVA; significance is indicated as ns, not significant, ^*^0.0079, ^***^0.0002, ^****^<0.0001. Reference isolate *L. tropica* strains (MHOM/AZ/1974/SAF-K27) and *L. infantum* strains (MHOM/TR/2006/CBU20), VL, visceral leishmaniasis group; CL, cutaneous leishmaniasis group; PRDX1/2, Peroxidoxin 1 and 2, OPB, Oligopeptidase B; M32, Metallo-peptidase, Clan MA(E), family M32; COX IV, Cytochrome C Oxidase subunit IV; SCOT, Succinyl-CoA:3-ketoacid-coenzyme A transferase; PK, Pyruvate kinase.

The genes previously associated with viscerotropism in *L. tropica* include Peroxidoxin 1/2 (LMJF_23_0040), Oligopeptidase B (LMJF_09_0770), Metallo-peptidase (Clan MA(E), M32 family protein) (LMJF_33_2540), Pyruvate kinase (LMJF_35_0020), Succinyl-CoA:3-ketoacid-coenzyme A transferase (LMJF_33_2340), and COX4 (LMJF_12_0670) (Dinç, 2018). In this study, mRNA levels of genes associated with viscerotropism were compared across cutaneous leishmaniasis (CL) isolates, visceral leishmaniasis (VL) isolates, reference *L. tropica*, and reference *L. infantum* groups; fold-change values were normalized to reference *L. tropica* = 1 (Figure 2). No significant differences were detected in the mRNA expression of the examined viscerotropic genes between CL isolates and reference *L. tropica*. By contrast, the VL isolates exhibited significant increases across multiple genes. Owing to high sequence homology, Peroxiredoxin-1 and Peroxiredoxin-2 could not be distinguished at the transcript level and were evaluated jointly; this combined expression was 17-fold higher in the VL group relative to the reference (*p* < 0.0001). The greatest increase was observed for COX4, with a 21-fold elevation (*p* < 0.0001). In addition, Metallo-peptidase, Clan MA(E) M32 family protein, Oligopeptidase B, and Succinyl-CoA:3-ketoacid-CoA transferase showed significant increases of approximately 3.1–6.1-fold (*p* < 0.05) in the VL group. No significant difference was observed for Pyruvate kinase in the VL group. In the comparative reference analysis, when reference *L. infantum* was evaluated alongside reference *L. tropica*, Peroxiredoxin-1/2 mRNA levels were 9.16-fold higher and Oligopeptidase B was 9.64-fold higher, whereas changes in the remaining genes were limited to ~1.37 to 1.84-fold. Overall, this pattern indicates that CL isolates share a similar expression profile with reference *L. tropica*, whereas VL isolates display marked upregulation of genes linked to oxidoreduction and protein processing.

### 3.3 Gene-level analysis of visceralized *Leishmania tropica*

Targeted next-generation sequencing (NGS) was used to interrogate coding regions of seven candidate genes implicated in viscerotropism and revealed a group-distinctive variant pattern (Table 3). Because of the high sequence homology between Peroxiredoxin-1 and Peroxiredoxin-2, a single representative sequence was deposited in GenBank (OQ689280). Across this locus, no discriminatory variants were detected among VL isolates, CL isolates, and the reference *L. tropica* strain. For Oligopeptidase B, the sequence was deposited in GenBank (OQ689281). At this locus, amino-acid–altering variation showed a distribution that was largely confined to CL isolates and the reference strain, whereas such changes were not observed in the VL isolates. Likewise, for the M32 metallo-peptidase (Clan MA(E), Family M32), deposited in GenBank (OQ689282), the overall pattern suggested that certain amino-acid substitutions were present in CL/reference strains but not in VL isolates; in contrast, VL isolates tended to display a higher degree of homozygosity for several synonymous substitutions relative to CL and reference strains. The COX4 sequence was deposited in GenBank (OQ689285). Variants detected in this gene showed a group-level difference in zygosity, with VL isolates more frequently exhibiting homozygous states, while CL isolates and the reference strain more commonly exhibited heterozygous profiles. Similarly, the gene encoding succinyl-CoA:3-ketoacid-CoA transferase, deposited in GenBank (OQ689284), displayed a comparable group separation: several synonymous variants tended to be homozygous in VL isolates, whereas the corresponding changes remained heterozygous in CL isolates and the reference strain. Finally, pyruvate kinase, deposited in GenBank (OQ689283), also demonstrated a broader tendency toward homozygosity in VL isolates compared with CL isolates and the reference strain (Table 3). Collectively, the variant landscape summarized in Table 3 does not indicate a single VL-specific missense “signature.” Rather, certain amino-acid substitutions appear restricted to CL/reference strains, whereas VL isolates show a tendency toward homozygosity for multiple synonymous changes. Taken together with the expression findings, these results support the interpretation that viscerotropism may reflect not only protein-sequence alterations but also expression dynamics and potential regulatory effects.

**Table 3:** Summary of missense mutations identified through targeted next-generation sequencing (NGS) in *Leishmania tropica* isolates from VL and CL patients, highlighting differences potentially associated with disease tropism.

Viscerotropism associated genes	Reference *L. tropica*	CL	VL
Peroxidoxin 1	–	–	–
Peroxidoxin 2	–	–	–
Oligopeptidase B	c.1031A > G (p. Asp344Gly) c.1306C > G (p. Pro436Ala)	c.1031A > G (p. Asp344Gly) c.1306C > G (p. Pro436Ala)	–
Metallo-peptidase, Clan MA (E), M32 family protein	c.169G > T (p. Ala57Ser)	c.169G > T (p. Ala57Ser)	–
Cytochrome C Oxidase subunit IV	–	–	–
Succinyl-CoA:3-ketoacid-coenzyme A transferase	–	–	–
Pyruvate kinase	c.1362G > A (p. Glu454Lys)	c.1362G > A (p. Glu454Lys)	c.1362G > A (p. Glu454Lys)

Reference L. tropica (MHOM/AZ/1974/SAF-K 27).

## 4 Discussion

Viscerotropic *L. tropica* is characterized by its capacity to initiate visceral or systemic infections in humans. Numerous studies have investigated the pathogenesis and clinical features of viscerotropic leishmaniasis caused by *L. tropica*. Although *L. tropica* is predominantly recognized as the causative agent of CL, its involvement in visceral leishmaniasis has been reported, challenging conventional understanding. The first evidence of visceral leishmaniasis caused by *L. tropica* was reported by Aljeboori and Evans, who demonstrated that a strain isolated from a patient diagnosed with VL in Iraq exhibited biochemical characteristics closely resembling those of *L. tropica* (Aljeboori and Evans, 1980). Similarly, Schnur et al. identified *L. tropica*-associated strains with viscerotropic properties through isoenzyme analysis of human isolates from Saudi Arabia (Schnur et al., 1981). In the study by Mebrahtu et al., *Leishmania* strains isolated from two patients diagnosed with VL in Kenya were characterized by isoenzyme profiling and molecular analysis, confirming their identity as *L. tropica* and highlighting their potential for visceral tropism (Mebrahtu et al., 1989). Subsequently, among eight U.S. military personnel returning from the Gulf War diagnosed with VL, six isolates were identified as *L. tropica* using isoenzyme analysis (Magill et al., 1993). A case study involving a patient initially presenting with skin lesions and subsequently developing VL nine months later confirmed *L. tropica* as the causative species via PCR analysis of skin, blood, and bone marrow samples (Alborzi et al., 2008). Recent studies employing molecular techniques frequently confirm *L. tropica* as a VL-causing agent. For instance, sequencing of splenic puncture samples from VL patients in endemic regions of Iran identified *L. tropica* with 99.9% certainty (SARKARI et al., 2016). Further, genetic analyses of the internal transcribed spacer-1 (ITS-1) region confirmed VL-associated *L. tropica* isolates (Ghatee et al., 2018). Genetic investigations revealed that expression of the viscerotropic leishmaniasis antigen gene (VTL) was elevated threefold in VL-causing *L. tropica* compared to *L. infantum* (Nafchi et al., 2016). It remains necessary to clarify whether the VL-causing *L. tropica* represents a subpopulation of the species or reflects a broader regional or global genetic pool, and whether it engages in genetic exchange with other species. Although recombination has been demonstrated among *Leishmania infantum* strains, studies from sympatric endemic foci where *L. infantum* and *L. tropica* co-occur (e.g., Morocco) have not detected genetic exchange (gene flow) between these two species (El Mazini et al., 2023). This finding suggests that the pronounced diversity observed in the *L. tropica* population structure (Salloum et al., 2020) likely results from intraspecific clonal expansion, limited recombination, or geographic isolation, rather than hybridization with other *Leishmania* species. North African regions, particularly Morocco, constitute major endemic areas with high genetic diversity for *L. tropica* (Talimi et al., 2024). Advanced genomic analyses conducted in this region have revealed the complexity of the population structure: comparative genome analysis identified, in addition to a core group, two highly divergent strains (M3015 and Ltr_16) that are phylogenetically distinct from both the current *L. tropica* reference genome and the core group (Talimi et al., 2024). This observation indicates that genetic heterogeneity is not confined to widespread strains but also encompasses rare, evolutionarily deep subpopulations.

Although the present study was intentionally designed to prioritize parasite-intrinsic molecular determinants of viscerotropism in *L. tropica*, we acknowledge that the epidemiology and clinical expression of leishmaniasis are shaped by a broader set of contextual drivers that were not systematically modeled here. At the environmental/ecological level, climate variables (temperature, rainfall, humidity) and broader climate-change trends can modify sand fly survival, density, and transmission seasonality, while land-use change and micro-environmental housing conditions (e.g., structural cracks, animal shelters, and sanitation) can create or shift vector resting/breeding sites and alter human–vector contact patterns (Valero and Uriarte, 2020; Paz, 2024; Senanayake et al., 2024). At the host immunological level, disease containment versus progression is strongly influenced by host genetic background and immune polarization (e.g., Th1/Th2/Th17 balance), as well as macrophage effector mechanisms such as reactive oxygen species and nitric oxide production that determine intracellular parasite control (Costa-Da-silva et al., 2022; Morales-Primo et al., 2024). At the socioeconomic and behavioral level, poverty, crowded or substandard housing, occupational exposure, population mobility, and health-system access can all influence transmission intensity, timely diagnosis/treatment, and ultimately observed clinical phenotypes at the population level (Valero and Uriarte, 2020; Awab, 2023). These interacting determinants may contribute to heterogeneity in parasite burden, immune pressure, and within-host environments that shape parasite adaptation *in vivo*. Consistent with our study’s scope, we focused on parasite genetic/expression signatures in clinically derived isolates, while recognizing that *Leishmania* gene expression can also be modulated by parasite genomic plasticity (including gene dosage/CNV) and by host- and environment-driven selection pressures (Iantorno et al., 2017; Glans et al., 2021). Future work integrating parasite multi-omics with structured environmental exposure metrics and host immunophenotyping across geographically diverse cohorts will be important to place parasite-intrinsic signals within the full eco–immuno–social framework governing *L. tropica* infection outcomes. From an evolutionary perspective, increasing evidence suggests that visceral competence in some *L. tropica* lineages reflects adaptive diversification rather than a fixed species-level trait*. L. tropica* shows marked genome plasticity (e.g., aneuploidy and gene-dosage/CNV shifts) and signatures of genetic exchange/mosaic ancestry, which can rapidly reshape multigenic programs relevant to stress tolerance, intracellular survival, and tissue-specific fitness. Ecological flexibility and shifting transmission networks (including in Türkiye and neighboring regions) may further promote selection in distinct host/vector and environmental contexts. Experimental observations also support biological plausibility, indicating that some *L. tropica* backgrounds can disseminate and persist in visceral organs, consistent with a polygenic and context-dependent model of visceralization that aligns with the stress-adaptation signatures highlighted by our candidate-gene findings.

This study examined seven candidate genes previously implicated in *L. tropica* viscerotropism based on prior proteomic evidence, and qRT-PCR analyses demonstrated significantly increased mRNA expression for six genes (COX4, PRDX1/2, M32 metallo-peptidase, Oligopeptidase B, and SCOT) in VL-derived isolates, whereas pyruvate kinase did not show a significant change. Among these genes, the most striking finding was the 21-fold increase in COX4 mRNA expression in *L. tropica* isolates obtained from VL patients. This observation supports previous reports suggesting that elevated Cytochrome C oxidase expression is associated with higher virulence and resistance to antimonial therapy in *Leishmania* species (Cardenas et al., 2015; Hajjaran et al., 2015). Cytochrome C oxidase, located in the inner mitochondrial membrane, serves as the terminal component of the electron transport chain and plays a critical role in ATP production. The pronounced overexpression of this subunit likely reflects the heightened energy demands of the visceral milieu, possibly driven by rapid intracellular proliferation and the requirement to overcome the host’s immune responses. Studies in *L. major* have shown that COX4 (LmCOX4) expression is regulated by a protein called LACK, which governs the parasite’s thermotolerance and virulence; disruption of LACK leads to reduced LmCOX4 levels, impaired mitochondrial function, and decreased ATP production. Restoring LmCOX4 expression in these LACK-deficient parasites rescues their ability to tolerate mammalian temperatures and improves their infectivity in macrophages (Cardenas et al., 2015). The 21-fold increase observed in visceralized *L. tropica* isolates is thus consistent with the broader mechanism utilized by various *Leishmania* species, including *L. donovani*, to adapt and replicate within visceral organs (Cytochrome c oxidase subunit IV - Leishmania donovani | UniProtKB | UniProt, n.d.).

Another key finding of this study was the 17-fold increase in Peroxiredoxin 1 and 2 mRNA expression in VL isolates compared to reference *L. tropica* strains. Peroxiredoxins are a family of antioxidant enzymes that protect cells against oxidative stress by neutralizing reactive oxygen species (ROS), which are abundantly generated by the host immune system during infection (Castro et al., 2011; Hajjaran et al., 2015). In line with these results, Hajjaran *et al.* reported that increased Peroxiredoxin expression supports parasite survival in visceral organs by mitigating macrophage-derived oxidative stress (Hajjaran et al., 2015). Studies in *L. infantum* and *L. donovani* further emphasize the importance of mitochondrial peroxiredoxin in infection, showing that its absence or reduction significantly compromises parasite survival in murine models and increases sensitivity to oxidative damage (Isaza et al., 2008; Castro et al., 2011). Notably, even a peroxidase-inactive version of Peroxiredoxin can restore infectivity, suggesting an additional role for this enzyme as a molecular chaperone (Castro et al., 2011). In the present study, the expression levels of Peroxiredoxin 1 and 2 genes in reference *L. infantum* strains were 9.16-fold higher compared to *L. tropica* strains, indicating a conserved antioxidant defense mechanism among *Leishmania* species. However, it must be considered that the expression profiles of redox-related genes such as Peroxiredoxin 1 and 2, which play a crucial role in the oxidative stress response, may be influenced by *in vitro* culture conditions, and therefore the potential for culture-derived artifacts should be acknowledged. On the other hand, findings demonstrating increased susceptibility to oxidative stress in spontaneously healing cutaneous leishmaniasis cases further underscore the protective and adaptive roles of enzymes belonging to the Peroxiredoxin family in visceral infections (Sarkar et al., 2012). These observations suggest that the aforementioned genes might have critical functions in the development of visceral tropism and in enabling parasite survival against host defense mechanisms. Additional genes that showed significant increases in mRNA expression in VL *L. tropica* isolates included Metallo-peptidase (Clan MA(E), M32 family) (6.1-fold), Oligopeptidase B (3.9-fold), and Succinyl-CoA:3-ketoacid-coenzyme A transferase (SCOT) (3.1-fold). Metallo-peptidases participate in various processes critical to parasite survival and proliferation—such as nutrient acquisition, protein processing, and immune evasion (Isaza et al., 2008; Alvarez et al., 2012). Consequently, the upregulation of this M32 family protease may bolster *L. tropica* adaptation within visceral organs. Oligopeptidase B, a serine protease associated with macrophage infection and intracellular survival (Swenerton et al., 2011; Goyal et al., 2014), was also more highly expressed in VL isolates. Notably, reference *L. infantum* exhibited a 9.64-fold increase in Oligopeptidase B gene expression compared to reference *L. tropica*, possibly highlighting a species-specific significance for this enzyme in visceralization. SCOT plays a key role in ketone body metabolism, thereby furnishing the parasite with an alternative energy source in glucose-limited environments such as host macrophages (Ashrafmansouri et al., 2022). Taken together, the collective overexpression of these genes suggests a multifaceted suite of adaptive strategies that enables *L. tropica* to survive and proliferate in the visceral environment.

When VL-derived *Leishmania tropica* isolates were compared with the reference *Leishmania infantum* strain, a broadly comparable elevation in PRDX1/2 and Oligopeptidase B expression was observed, suggesting convergence on antioxidant and proteolytic programs that can mitigate oxidative stress and support intracellular persistence. This interpretation is consistent with host-side transcriptomic evidence from human macrophage-like cells, in which infection with the viscerotropic species *L. infantum* (relative to *L. major*/*L. tropica*) was associated with early enrichment of antioxidant/detoxification pathways—responses linked to reduced cellular oxidative stress and discussed as potentially relevant to visceralization. Notably, in that infection model, pathways implicated in visceralization displayed opposing regulation patterns between viscerotropic (*L. infantum*) and dermotropic (*L. major*/*L. tropica*) infections, underscoring that similar clinical outcomes may be achieved through distinct host–parasite interaction strategies (Diotallevi et al., 2024). In contrast, in our parasite-side comparison, the fold changes for Metallo-peptidase, Pyruvate kinase, SCOT, and COX4 in reference *L. infantum* were generally lower than those observed in VL-derived *L. tropica*, which may reflect species-specific evolutionary trajectories and niche-adaptation strategies. Overall, the prominence of antioxidant and peptidase activities across these comparisons supports a model in which oxidative-stress control is a key constraint shaping visceral competence, whereas the magnitude and composition of accompanying metabolic programs may differ across *Leishmania* lineages.

Viscerotropism in *L. tropica* involves the coordinated expression and functional modulation of multiple genes critical for parasite survival and proliferation in visceral tissues. Elevated expression levels of COX4 significantly enhance mitochondrial energy production and parasite fitness under visceral environmental stressors, such as fluctuating oxygen tensions and immune pressures (Kumar and Nylén, 2012; Sohrabi et al., 2013; Shaheen et al., 2021). Metallo-peptidase (Clan MA(E), Family M32) and Oligopeptidase B are implicated in protein processing and immune evasion, respectively, facilitating adaptation to internal organ environments by aiding nutrient acquisition and intracellular persistence (MEROPS - the Peptidase Database, n.d.; Swenerton et al., 2010; Salas-Sarduy et al., 2019; Barbosa et al., 2022). Notably, increased Peroxidoxin *1* and Peroxidoxin *2* expression contributes significantly to antioxidant defense mechanisms, crucial for mitigating oxidative stress imposed by heightened immune responses within visceral organs (Harder et al., 2006; Brígido et al., 2025). Pyruvate kinase and Succinyl-CoA:3-ketoacid-coenzyme A transferas*e* support essential metabolic flexibility, enabling utilization of alternative carbon sources and energy optimization in glucose-limited visceral niches (Rigden et al., 1998; Saunders et al., 2014; Mondal et al., 2020; Alotaibi, 2021). These genetic adaptations represent a multifaceted molecular strategy that could help in explaining *L. tropica*’s shift from cutaneous to visceral tropism, with important implications for the development of diagnostic markers and targeted treatments for viscerotropic leishmaniasis (Mebrahtu et al., 1989; Hajjaran et al., 2015; Nafchi et al., 2016). Clinical relevance and potential applications. Although the present work is not intended as a diagnostic study, the observed, concordant upregulation of COX4 and PRDX1/2 (with supportive increases in M32, OPB, and SCOT) suggests a compact molecular “stress-adaptation” signature that could be evaluated as an adjunct marker set in endemic settings—particularly in contexts where *L. tropica* is identified but the risk of visceral involvement is clinically uncertain. In principle, once validated in larger and geographically diverse cohorts, such a signature could be explored in two complementary ways: (i) as a laboratory-based panel measured in early promastigote cultures derived from clinical specimens, and/or (ii) as a targeted transcript readout that complements established species identification (e.g., ITS1 genotyping) and existing serological/clinical assessment, rather than replacing them (Nafchi et al., 2016; Ghatee et al., 2018). Importantly, any clinical utility would require rigorous prospective validation and assessment of performance across diverse parasite genetic backgrounds and host contexts. Therapeutic relevance and prioritization of targets. Several of the identified candidates are mechanistically plausible and therapeutically attractive because they are directly linked to intracellular survival constraints in visceral macrophage niches (oxidative stress, nutrient limitation, and mitochondrial fitness). In particular, OPB has been highlighted as a potential drug target in trypanosomatid diseases and has existing inhibitor-focused mechanistic work (Goyal et al., 2014; Motta et al., 2019). Likewise, M32-family metallocarboxypeptidases are trypanosomatid-associated enzymes and have been explored through inhibitor discovery efforts, strengthening their candidacy for parasite-selective intervention strategies (Frasch et al., 2012; Salas-Sarduy et al., 2019). These considerations underscore that the present transcript-level findings may help prioritize parasite pathways for downstream experimental validation and drug-target assessment. Concrete future directions and testable hypotheses. To address the key limitation of absent functional validation and to strengthen translational inference, future studies should directly test: (i) whether COX4 upregulation causally increases mitochondrial respiration/thermotolerance and enhances intracellular fitness in macrophage infection models(Cardenas et al., 2015); (ii) whether PRDX1/2 upregulation improves survival under oxidative burst conditions, including macrophage-derived ROS, and whether this effect is required for visceral persistence (Harder et al., 2006; Castro et al., 2011); and (iii) whether OPB and M32 activity modulates macrophage invasion, intracellular persistence, and immune evasion, including sensitivity to available or newly developed inhibitors (Goyal et al., 2014; Salas-Sarduy et al., 2019; Barbosa et al., 2022). In parallel, broader discovery-scale efforts should extend beyond the candidate panel to include established virulence and tropism determinants (e.g., GP63 and LPG-related pathways, as well as other stage- and niche-adaptation modules) to refine the molecular framework of *L. tropica* visceralization (McCall et al., 2013). In considering the catalytic activities underlying *L. tropica*’s visceralization, we propose that oxidoreductase and antioxidant functions are critical for maintaining the redox balance necessary for parasite survival, while hydrolase activities may facilitate nutrient acquisition or modulate host immune responses. Overall, these findings could help in identifying molecular pathways involved in *L. tropica* adaptation to visceral environments and may inform future therapeutic approaches against visceral leishmaniasis. Genetic mutation analysis of the seven candidate genes revealed no missense or nonsense mutations in Peroxidoxin 1 and 2, COX4, or Succinyl-CoA:3-ketoacid-coenzyme A transferase. However, Oligopeptidase B exhibited two missense mutations, and Metallo-peptidase showed one missense mutation. Oligopeptidase B, belonging to the S9 prolyl oligopeptidase family, is recognized as a significant virulence factor in *Leishmania* (Motta et al., 2019). Metallo-peptidases (M32 family) are exclusively expressed by trypanosomatids and not found in other eukaryotes (Frasch et al., 2012). The heterozygous mutations identified as specific to CL isolates—p. Asp344Gly and p. Pro436Ala in Oligopeptidase B, and p. Ala57Ser in the metallo-peptidase—point to potential contributions to cutaneous tropism and suggest that tissue lesion formation and remodeling during infection may be affected. However, without testing their potential effects on codon usage, mRNA stability, and translational efficiency, and in the absence of functional validation, any putative impact on protease activity and tissue remodeling remains inferential.

The study investigating genes associated with viscerotropism in *L. tropica* strains causing visceral leishmaniasis (VL) has several limitations that should be stated explicitly. First, the limited number of clinical isolates (*n* = 14) may restrict generalizability; therefore, validation in larger, geographically diverse cohorts is needed. Second, gene-expression analyses were performed primarily in promastigote cultures rather than in the clinically relevant amastigote stage; future studies should assess expression directly in tissue-derived amastigotes and/or controlled infection models. Third, although culture conditions were standardized and passaging was minimized, *in vitro* growth may not fully reproduce the visceral microenvironment, and validation beyond transcript-level readouts—including experimental infection models, protein-level/enzymatic activity measurements, and gene-editing–based functional assays (e.g., knockout/overexpression)—was beyond the scope of this work, thereby limiting causal inference. Fourth, the work was designed as a focused, hypothesis-generating candidate-gene study of seven previously implicated genes; thus, additional pathways and determinants of virulence/tropism (including surface factors such as GP63 and LPG-related genes) were not examined. Relatedly, broader environmental, immunological, and sociodemographic drivers—as well as the evolutionary dynamics of *L. tropica* across hosts/ecologies—were not explicitly modeled and warrant dedicated investigation. Fifth, inter-isolate variability may influence transcript-level readouts; accordingly, our findings should be interpreted as group-level signals rather than uniform effects across all individuals. Finally, because CNV can affect transcript abundance in *Leishmania*, a CNV contribution cannot be excluded; however, dedicated gDNA-based copy-number quantification was beyond the scope of this study and should be addressed in future work using ddPCR or genome-wide approaches.

## 5 Conclusion

This study identifies parasite-intrinsic molecular features associated with visceral tropism in *Leishmania tropica*. Compared with CL isolates and reference strains, VL-derived *L. tropica* isolates showed significant upregulation of COX4, Peroxiredoxin 1/2, M32 metallo-peptidase, Oligopeptidase B, and Succinyl-CoA:3-ketoacid-coenzyme A transferase, supporting coordinated enhancement of pathways linked to mitochondrial function, antioxidant defense, proteolysis, and metabolic adaptation that may facilitate persistence in visceral tissues. These findings advance understanding of *L. tropica* pathogenicity and may inform improved surveillance and the prioritization of testable diagnostic and therapeutic targets relevant to VL control. However, as conclusions are based on transcript-level changes within a focused candidate-gene panel, larger cohorts and functional validation (including protein-level confirmation and experimental models) are required to establish causality and refine clinical utility.

## Data Availability

The datasets presented in this study can be found in online repositories. The names of the repository/repositories and accession number(s) can be found in the article/Supplementary material.
