# Supplementary material for: Investigation of viscerotropism-associated genes in Leishmania tropica strains causing visceral leishmaniasis
Source: Front Microbiol. 2026 May 11;17:1831849. doi: 10.3389/fmicb.2026.1831849 (PMC13199171; doi:10.3389/fmicb.2026.1831849)
Supplement: Supplementary file 1 [file Table_1.DOCX]

**SUPPLEMENT MATERIAL**

**SUPPLEMENT TABLE 1.** Forward and reverse primers designed for NGS analysis of seven genes. The Ion Torrent PGM system supports read lengths up to 400 bp; however, optimal performance is achieved with amplicons around 200 bp, ensuring high-quality sequence data. The sequencing of the seven genes associated with viscerotropism was conducted using overlapping amplicons of approximately 200 bp. The primers listed in Table S1 are specific to these amplicons for each gene. Additionally, primers marked with "*" were also utilized in the mRNA expression analysis of the seven genes.

| **Species** | **Genes** | **Primers** | **Amplicon size (bp)** |
| --- | --- | --- | --- |
| *Leishmania tropica* | Peroxidoxin 1 and 2 | F: CTCTCCCAACGCTCTCCAC  R: GCGGAGAGGCTGATCTTCTT | 169 |
|  |  | **F:* CCAACGGCAGCTTCAAGAA  **R:* CGTACTCGCTGTCCATCGA | 183 |
|  |  | F: CTACCGCGGTCTCTTCATCA  R: GCCCTTCTTCCAGTTCGC | 163 |
|  |  | F: GTGGAGAAGCACGGCGAG  R: CTGACTCCTATGACACTCACA | 129 |
|  | Oligopeptidase B | F: GTTCTGTTAGTCTTTGAAGA  R: GTCCTCACCCTCCACGTAG | 187 |
|  |  | **F:* CTACGTGGAGGGTGAGGAC  **R:* TGTCGTCCTCCTTGATGTGC | 218 |
|  |  | F: CTACGCTGAGCACATATCGC  R: CACGTCACAGAAGGGTTTGC | 211 |
|  |  | F: GGCAAACCCTTCTGTGACG  R: CCTTGTCGGCGATGGTTTG | 148 |
|  |  | F: CCAAACCATCGCCGACAAG  R: CGGCTTGCCCATCACGTG | 136 |
|  |  | F: TGACCAAAGACGAGACGCT  R: GCACCATCTCCAGCGTATTG | 231 |
|  |  | F: CAATACGCTGGAGATGGTGC  R: GTCCACCATCATCGTCCAGA | 274 |
|  |  | F: AGTACTCCTCCCTTGCCAC  R: CTGAATGCTGAACTGCGGG | 187 |
|  |  | F: CCCGCAGTTCAGCATTCAG  R: GAAAAGGTGTTGCGCTTCGT | 141 |
|  |  | F: CGCAACACCTTTTCCGACTT  R: CCACCTTGAAGAGATCGGGA | 151 |
|  |  | F: ATGCGTCCCGATCTCTTCAA  R: GTTGCCCCACTCCTCCCA | 117 |
|  |  | F: TGGGAGGAGTGGGGCAAC  R: GAGAAGTGTCCGCTCTCCAT | 233 |
|  |  | F: ATGGAGAGCGGACACTTCTC  R: CTCCCTCGTCCCTTGTGAC | 185 |
|  | Metallo-peptidase, Clan MA (E), M32 family protein | F: ACCGCATTTTCTCTGAGCAC  R: GTGACTCCTCGCCCTTGG | 160 |
|  |  | **F:* CCAAGGGCGAGGAGTCAC  **R:* GTGAAGTCATTTGCGTTGCG | 271 |
|  |  | F: CGCAACGCAAATGACTTCAC  R: GATGCCCGGCTCGTACTC | 140 |
|  |  | F: GAGTACGAGCCGGGCATC  R: GTTTGTTGTGAGTCGGCAGT | 270 |
|  |  | F: AGATCTGGCACTTCGACACC  R: CTGAAACTCCGCAAAGAGGC | 260 |
|  |  | F: GACTACCTCACAACTCGCCT  R: GAGTACGTCGGGAAGTAGCC | 87 |
|  |  | F: GGCTACTTCCCGACGTACTC  R: GGTCATCCGTCTCGTACAGG | 181 |
|  |  | F: CCTGTACGAGACGGATGACC  R: GCGCGTTACAAGATGGTCTC | 240 |
|  | Cytochrome C Oxidase subunit IV | F: CCCCTTTGCCCAATCGAAAA  R: GTGCGTCTTCATCCAGCTTG | 215 |
|  |  | **F:* ACTGGACACGCACAACTACA  **R:* TCTCGAGGCGGTACTTGATC | 212 |
|  |  | F: GATCAAGTACCGCCTCGAGA  R: TGTCCTCAGCCAACTCGTTC | 197 |
|  |  | F: CAGGCGGAGATGAAGTACGT  R: GGCTACGGTCTGGAAGTCAT | 228 |
|  |  | F: GGCTACCAGAGAAGTACCACA  R: TGATGTTGCGGGTCGGAT | 207 |
|  |  | F: GAGCTCCTGGACGACGTC  R: CATCTGCCTGACCAGCGC | 195 |
|  |  | F: GCGCTGGTCAGGCAGATG  R: CGACAAAGAACTGACCGGAC | 147 |
|  | Succinyl-CoA:3-ketoacid-coenzyme A transferase | F: GTTTCTGACCACCATCTCGC  R: GATGCCATCCGTCATGTCC | 116 |
|  |  | **F:* GGACATGACGGATGGCATC  **R:* CGATGAGACGGGCGAGAC | 158 |
|  |  | F: GGTCTCGCCCGTCTCATC  R: CGTGTAGAAGGCAGGGATGC | 180 |
|  |  | F: CATCCCTGCCTTCTACACGG  R: GGCGGGGATGTTGAAGTTG | 250 |
|  |  | F: CTGCCCGCAACTTCAACAT  R: CCGCTACCAGAGACCGTTC | 184 |
|  |  | F: GTCGTACGAGGTGCCCAT  R: ATATAGTTCGCCGCCTCCG | 174 |
|  |  | F: CGGAGGCGGCGAACTATAT  R: GATACCCCAGTTCGCGAGG | 256 |
|  |  | F: CGGAGGCGGCGAACTATAT  R: GATACCCCAGTTCGCGAGG | 266 |
|  |  | F: GGCGGCCACATGAACCTG  R: AATGCAGGCCCGTCACAG | 220 |
|  |  | F: CTGTGACGGGCCTGCATT  R: GATCGGCACCCTCAGACC | 233 |
|  | Pyruvate kinase | F: CCGCCGTTTCTCAACTCATT  R: GTGACCCATGCGAGAAGTTC | 235 |
|  |  | **F:* GAACTTCTCGCATGGGTCAC  **R:* CAGGGTCTGTCGTCACGTAG | 191 |
|  |  | F: CTACGTGACGACAGACCCT  R: GTGTGCGCGTTAGTCACC | 189 |
|  |  | F: ACACGATCTCTGACCGGC  R: TGGTGGTTCTCGATCTTGCA | 223 |
|  |  | F: TGCAAGATCGAGAACCACCA  R: GGGTTGTACGTCATGCTCTC | 209 |
|  |  | F: GTCTTCAACGGTGCGGATTG  R: AAACAGCCTCCTCAGCACTC | 184 |
|  |  | F: GAGTGCTGAGGAGGCTGTTT  R: ACGCCTTGTGTGATGTTCAG | 189 |
|  |  | F: GCCAGCTGAACATCACACAA  R: CGAGAAGAATGCGTGTCTGG | 204 |
|  |  | F: TGTGCAGACTGGTGACTACA  R: GAATGGTGGGGTTGACGAAC | 189 |
|  | 18S rRNA | **F:* GAGTTGAAAAGGCGTTACGG | 78 |
|  |  | **R:* GCCTTTGAGGGGTTTAGTGC |  |
| *Leishmania infantum* | Peroxidoxin 1 and 2 | **F:* CTCTCCCAACGCTCTCCAC  **R:* CGTACTCGCTGTCCATCGA | 169 |
|  | Oligopeptidase B | **F:* GTTCTGTTAGTCTTTGAAGA  **R:* GTCCTCACCCTCCACGTAG | 187 |
|  | Metallo-peptidase, Clan MA (E), M32 family protein | **F:* CCAAGGGCGAGGAATCAC  **R:* GAAGTCATTTGCTTTGCG | 160 |
|  | Cytochrome C Oxidase subunit IV | **F:* GCTGGACACGCACAACTATA  **R:* TCTCGAGGCGGTACTTGATC | 215 |
|  | Succinyl-CoA:3-ketoacid-coenzyme A transferase | **F:* GGACATGACGGACGGCATC  **R:* CGATGAGACGGGCGAGAC | 116 |
|  | Pyruvate kinase | **F:* GAACTTCTCGCATGGGTCAC  **R:* CCATCCAATCGGTAGTAGCG | 235 |
|  |  |  |  |
